# Correlation between risk factors that influence the development of children's language

**DOI:** 10.1590/2317-1782/e20240131en

**Published:** 2025-01-27

**Authors:** Marianna Momoe Nanakuma Matsumoto, Heloisa Adhmann Ferreira, Isabelly Bueno Araujo, Daniela Cardilli-Dias, Daniela Regina Molini-Avejonas

**Affiliations:** 1 Faculdade de Medicina, Universidade de São Paulo – USP - São Paulo (SP), Brasil.

**Keywords:** Speech, Language and Hearing Sciences, Risk Factors, Language Development, Participant Health Questionnaire, Child Health, Primary Health Care

## Abstract

**Purpose:**

To identify the most significant risk factors for child development through the application of two risk protocols, namely, the Protocol for the Identification of Risk Factors for Language and Speech Disorders (PIFRAL) and the Language Development Protocol (PDL).

**Methods:**

A retrospective study was carried out with 194 children aged 0 to 5 years and 11 months who were participants of primary health care (PHC) in the municipality of São Paulo, Brazil, from 2016 to 2020. The database was thoroughly analyzed using R software, and the most relevant risk factors were correlated through statistical analysis, generating altered and unaltered PDL results. Altered PDL results in the presence of one or more altered axes.

**Results:**

Of the 194 participants, 62.4% had altered PDLs, and the risk factors that were most common in this group were male gender; being white; having a family socioeconomic level of upper middle, lower middle or low class; having a level of education up to high school; having a child with an altered temperament and having a mother with an altered temperament.

**Conclusion:**

This research has achieved its goals, first, by correlating the PIFRAL and PDL protocols and second, by showing some of the risk factors for child development and their implications for language acquisition.

## INTRODUCTION

Language is the main method that humans use for relaying information; consisting of words conveyed by speech, writing, or gestures. Language is the conceptual processing of communication^(1).^ Language development is a fundamental step for the individual to relate to society and the manifestation of language in its oral form is, within child development, one of the most expected milestones, since it allows greater communicative flexibility as the most accepted social means of interaction^(2)^.

### Biological and environmental factors

The combination of biological, cognitive, psychosocial and environmental factors is highly important for the child’s learning process^(3)^. The risk factors associated with biological and environmental factors such as low parental education level, relationship by blood, family history presenting speech-related issues and inadequate stimulation may result in speech and language delay^(4)^.

Another significant risk factor is low socioeconomic status. Children with developmental disadvantages are more vulnerable to language-related disorders^(5)^.

A study conducted in 2017, at the Speech-Language Pathology and Audiology Investigation Laboratory in Primary Health Care (LIFAPS) of the School of Medicine of Universidade de São Paulo found that the main risk factors in children with language and child development disorders were: existing family history, being born prematurely or under-weight, low maternal education level, long hospitalization time, and low socioeconomic status^(6)^.

The first 6 years are crucial for language development. Children with little exposure, in terms of quality and quantity, to linguistic stimuli tend to manifest delays in the development of important language components such as linguistic semantic, morphosyntactic, semantic, pragmatic, lexical aspects, etc. Oral language paves the way for the child to begin discovering and exploring the world, objects and the people around them^(7)^.

Quality time spent between the parents and their children poses as a source for children well-being. Activities and experiences carried out as a group by a family enable the children to express their thoughts and feelings freely. This in turn contributes to their quality of health and consequently lowers their risk factors^(8)^. Time well spent between parents and their children generates greater success^(9)^.

In addition to these factors, current research indicates that the male gender can be one of the risk factors for language disorders, due to the slower maturation of the nervous system in boys^(10)^.

### The need for speech therapy

Attention to changes in language levels is necessary, as well as to the risk factors that may lead to changes in these levels. Speech therapy, as the science of communication, has a fundamental role in the integration of health promotion and disease prevention practices. Therefore, speech therapy is recommended in any case of speech and language delay for proper diagnosis and treatment. Children should be monitored carefully for delayed milestones, especially regarding speech and care should be sought if a delay is observed^(1)^.

The Primary Health Care (PHC) is the participant's gateway into the health system. It sets the flow of health care for that user in the network as it aims to enhance the guarantee of comprehensiveness, continuity and efficiency of the health system. In addition, PHC must be able to maintain a bond with these users, providing continuity of care (health promotion and disease prevention, among others), even if they are also being cared for at other points of care in the network^(11)^. Primary Care (PC) contributes to child development as it follows them since before birth. It is the responsibility of the healthcare teams to monitor in prenatal care, puerperal (birth-related) visits, immunization, growth and development consultations, and so on, favoring the bond and the early identification of issues (through early screening) that need to be regularly and systematically followed-up^(12)^.

Speech-language intervention in children’s language development issues generates possibilities for improving language skills. An intervention must be considered as a necessity for children with developmental disorders, sensory or intellectual deficits, neurological disorders or disorders specifically related to one or more linguistic systems. It also contributes to cases with a history of organic, affective or environmental factors. A good assessment tool along with family participation in the intervention process ensures better results for the development of children's language^(13)^. Therefore, early intervention is one of the possibilities for the speech therapist.

### Speech-language screening

To this end, the type of evaluation has been increasingly discussed comprising its elements, objectives and ways of application. One of the tools that can be used for a quick screening is the translated version protocol “How Does Your Child Hear and Talk?”. This protocol is named Protocol for Language Development (PDL) and it is a version adapted and translated into Portuguese by Molini-Avejonas (2017) of the original produced by the American Speech-Language-Hearing Association (ASHA) and it is validated and used as a screening tool for children from 0 to 5 years old^(10)^.

The PDL is a checklist for speech, language and hearing development. It covers the ages: birth to one year, one to two years, two to three years, three to four years, and four to five years. The checklist is used as a screening tool for health professionals to determine if a child is on track or they may need extra help^(14)^. It was answered by parents/guardians as part of this research.

Another important tool is the risk protocol named Protocol for the Identification of Risk Factors for Language and Speech Disorders (PIFRAL). PIFRAL was developed in Brazil in 2013 based on risk factors to communication; it is a form containing 29 questions directed at parents/guardians. The questions are aimed at the sociodemographic profile (age, gender, declared race and child education level; age, parental education level and parents’ profession, place of residence), family life (number of siblings, birth order, twins, time spent with children, language used at home), prenatal, perinatal and postnatal complications as well as temperament of the child^(15)^.

Both these protocols are highly useful tools to help health professionals such as speech therapists working with children in Primary Health Care to quickly and assertively identify their language and speech disorders as soon as possible in order to start immediate care or as it is most common to form that bond that will provide them much needed continuity of care.

### Rationale

This research will provide health professionals and in particular speech therapists, with important risk factors to consider when they are actively screening children for speech-related disorders in Primary Health Care.

### Learning objectives

Firstly, this research aims at correlating the results found in the PDL with the answers obtained in the PIFRAL to identify the most significant risk factors for child development, above all language disorders. Secondly, our objective is to interpret the results found. Thirdly, we aim at providing research that will help formulate public policies for the population in question, while focusing on prevention, promotion, and early intervention. In that sense, this research has a local context.

## METHODS

This study was approved by the Ethics and Research Committee under the Authorization number: 2,437,351. Firstly, the Informed Consent Form (ICF) was applied to all participants in this research.

Data from the two applied risk factor protocols for infant language development (PIFRAL and PDL) were analyzed. One hundred and ninety (194) children who underwent care at the LIFAPS in the years 2016 to 2020 of up to 5 years and 11 months of age were considered. These participants were being treated for speech and language related disorders as well as audiology.

### Step-by-step

To explain in detail, we have organized an step-by-step description of the research methodology:

1. Speech therapy protocols that were applied from 2016 to 2020 in Primary Health Care for screening changes in child development were analyzed. Firstly, we applied the PDL.
2. For this study, participants aged up to 5 years and 11 months (early childhood) were selected.
3. Exclusion and inclusion criteria established, explained in detail further in this section.
4. Total participants screened: 321 -> excluded for not meeting the criteria: 127 -> TOTAL participants: 194
5. Out of the 194 participants after the PDL application -> 121 presented altered and 73 non-altered results.
6. All the participants that have PDL were correlated with the questions of the PIFRAL (child's gender; race; complaint type; time interval between the first speech-language pathology complaint and the child's age; family history; maternal education level; paternal education level; maternal age; time parents spend with their children; mother's temperament; father's temperament; child's temperament; socioeconomic status; pre- and postnatal complications; use of drugs, medication, alcohol and/or tobacco; prematurity/underweight; hospitalization; diagnosed disease; witnessed and/or violence endured).

### Inclusion criteria and exclusion criteria

Inclusion criteria: participants aged up to 5 years and 11 months and informed Consent Form (ICF) signed by parents/guardians.

Exclusion criteria: not properly filled out protocols, PDL and PIFRAL.

After analyzing the sample, the participants who did not meet the above-mentioned criteria were excluded.

### PDL

The PDL results were analyzed in two axes according to the child's age group: “Listening and understanding” and “Speaking”. The result was considered “altered” when the participant presented over 50% negative answers in at least one of the two axes. Alterations in both axes were not needed for the result to present as altered.

### PIFRAL

The following variables were selected and analyzed in contingency tables, correlating with the PDL result: sociodemographic questions, family life, prenatal, perinatal and postnatal care and temperament of the child.

### Data analysis

Afterwards, another set of data was analyzed: the time interval between the point in which the parents/guardians sought the speech therapy service and the first complaint (age of the child minus the age at the first complaint). To analyze these variables, a group of participants (N = 20) was excluded since they did not mention the age at which the speech-language complaint first appeared (time interval zero), as well as children who were born with hearing loss and/or any syndrome, and the time interval was 0 and/or 1 month of discovery of the condition (n = 5) and (n = 1), respectively. The remaining data resulted in a scatterplot which shows that the longer the parents took to look for help, the older the child was.

As a result of the scatterplot, firstly we compared data with the maternal education level and then the paternal education level, to analyze the health literacy of the participant's family.

### R software

The data was entered and analyzed by using the R software, version 4.1.1 (R Core team). It is friendly, easy-to-use and free open-source software, therefore available to all faculty members and student body.

### Qualitative and quantitative variables

For the analysis of qualitative variables, Pearson's chi-square test and the comparison of independence was used. The results were presented in contingency tables. For situations in which the chi-square test statistic was obtained, and the probability of finding a test statistic value greater than or equal to the result found, represented by p-value; the cutoff value of α=0.05 was used.

On the other hand, quantitative variables were analyzed in two manners: firstly, the two risk factors from the PIFRAL and the PDL were correlated in order to quantify the strength of this relation. Secondly, we performed the regression of the data in order to generate scatterplots with Pearson's linear correlation coefficient. The generation of the scatterplots was important in order to identify whether there is a gradual variability between the data sets, whether this variation is predominantly ascending or descending and whether a linear trend is assumed.

## RESULTS

Out of the 121 participants analyzed in this study that presented altered PDL, the prevalence was male (46.9%) in relation to the total (62.4%). The greatest demand for speech therapy services found was declared as Caucasian (68.0%) (Table 1). Most of the maternal and paternal education level answers were high school (Table 2). We have observed that there is an association between speech complaint and altered PDL of 49.5% in relation to the total of 74.2% (Table 3).

**Table 1:** Declared race

**PDL**	**Declared race**					**Total**
	**Asian**	**White**	**Black**	**NR**	**Multiracial**	
Altered	8 (4.1%)	88 (45.4%)	5 (2.6%)	4 (2.1%)	16 (8.2%)	121 (62.4%)
Non-altered	5 (2.6%)	44 (22.7%)	8 (4.1%)	1 (0.5%)	15 (7.7%)	73 (37.6%)
Total	13 (6.7%)	132 (68.0%)	13 (6.7%)	5 (2.6%)	31 (16.0%)	194 (100.0%)

Caption: PDL = Language Development Protocol; NR = Not reported

**Table 2:** Parents education level

**PDL**	**Maternal education level**									**Total**
	**EFC**	**EFI**	**EM**	**EMC**	**EMI**	**ES**	**ESC**	**ESI**	**NR**	
Altered	4 (2.1%)	6 (3.1%)	5 (2.6%)	46 (23.7%)	7 (3.6%)	1 (0.5%)	39 (20.1%)	10 (5.0%)	3 (1.5%)	121 (62.4%)
Non-altered	2 (1.0%)	2 (1.0%)	1 (0.5%)	25 (12.9%)	4 (2.1%)	2 (1.0%)	30 (15.5%)	5 (2.6%)	2 (1.0%)	73 (37.6%)
Total	6 (3.1%)	8 (4.1%)	6 (3.1%)	71 (36.6%)	11 (5.7%)	3 (1.5%)	69 (35.6%)	15 (7.7%)	5 (2.6%)	194 (100.0%)
**PDL**	**Paternal education level**									**Total**
	**EFC**	**EFI**	**EM**	**EMC**	**EMI**	**ES**	**ESC**	**ESI**	**NR**	
Altered	13 (6.7%)	7 (3.6%)	3 (1.5%)	38 (19.6%)	7 (3.6%)	4 (2.1%)	32 (16.5%)	9 (4.6%)	8 (4.1%)	121 (62.4%)
Non-altered	1 (0.5%)	4 (2.1%)	2 (1.0%)	28 (14.4%)	1 (0.5%)	1 (0.5%)	27 (13.9%)	4 (2.1%)	5 (2.6%)	73 (37.6%)
Total	14 (7.2%)	11 (5.7%)	5 (2.6%)	66 (34.0%)	8 (4.1%)	5 (2.6%)	59 (30.4%)	13 (6.7%)	13 (6.7%)	194 (100.0%)

Caption: PDL = Language Development Protocol; EFC = Complete primary education; EFI = Incomplete primary education; EM = Secondary education; EMC = Complete secondary education; EMI = Incomplete secondary education; ES = Higher education; ESC = Complete higher education; ESI = Incomplete higher education; NR = Not reported

**Table 3:** Type of complaints

**PDL**	**Type of speech-language complaint**										**Total**
	**H**	**S**	**SH**	**SL**	**SOM**	**S**	**L**	**OM**	**No**	**NR**	
Altered	3 (1.5%)	96 (49.5%)	3 (1.5%)	2 (1.0%)	2 (1.0%)	1 (0.5%)	5 (2.6%)	1 (0.5%)	0 (0.0%)	8 (4.1%)	121 (62.4%)
Non-altered	1 (0.5%)	48 (24.7%)	3 (1.5%)	0 (0.0%)	2 (1.0%)	5 (2.6%)	7 (3.6%)	1 (0.5%)	1 (0.5%)	5 (2.6%)	73 (37.6%)
Total	4 (2.1%)	144 (74.2%)	6 (3.1%)	2 (1.0%)	4 (2.1%)	6 (3.1%)	12 (6.2%)	2 (1.0%)	1 (0.5%)	13 (6.7%)	194 (100.0%)

Pearson's Chi-squared test data: Table X-squared = 12.587. df = 9. p-value = 0.1822

Caption: PDL = Language Development Protocol; H = Hearing; S = Speech; SH; Speech and hearing; SL = Speech and language; SOM = Speech and motor impulse of face and mouth; S = Stuttering; L = Language; OM = Orofacial motricity (Motor impulse of face and mouth); No = No complaint; NR = Not reported

**Figure 1:**
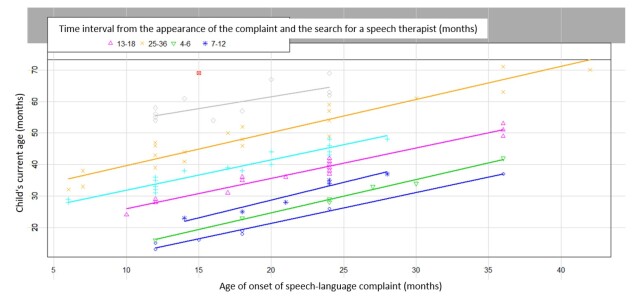
Time interval

The average time interval, in months, for children from 0 to 5 years and 11 months is equal to 20.10 (Figure 1).

Another question addressed in the protocol was the temperaments of the mother, father and child (Table 4). The altered temperament of the children was noteworthy. When analyzing only the altered PDL and comparing its correlation with the temperament of the child and the parent, we have observed statistically relevant numbers for altered maternal temperament, and altered child temperament (21.5%) in relation to the total in the vertical (28.1%).

**Table 4:** Temperaments

**PDL**	**Child's temperament**				
	**Altered**	**Non-Altered**	**NR**	**Total**	**p-value**
Altered	66 (34.0%)	48 (24.7%)	7 (3.6%)	121 (62.4%)	-
Non-Altered	39 (20.1%)	33 (17.0%)	1 (0.5%)	73 (37.6%)	-
Total	105 (54.1%)	91 (41.8%)	8 (4.1%)	194 (100.0%)	2.869
**PDL**	**Mother's Temperament**				
	**Altered**	**Non-Altered**	**NR**	**Total**	**p-value**
Altered	34 (17.5%)	81 (41.8%)	6 (3.1%)	121 (62.4%)	-
Non-Altered	32 (16.5%)	36 (18.6%)	5 (2.6%)	73 (37.6%)	-
Total	66 (34.0%)	117 (60.3%)	11 (5.7%)	194 (100.0%)	5.112
**PDL**	**Father's temperament**				
	**Altered**	**Non-Altered**	**NR**	**Total**	**p-value**
Altered	27 (13.9%)	79 (40.7%)	15 (7.7%)	121 (62.4%)	-
Non-Altered	22 (11.3%)	42 (21.6%)	9 (4.6%)	73 (37.6%)	-
Total	49 (25.3%)	121 (62.4%)	24 (12.4%)	194 (100.0%)	4.625

Caption: PDL = Language Development Protocol; NR = Not reported

Regarding pre, peri and/or postnatal complications, only the diagnosed disease obtained statistically relevant values, although it is important to emphasize that the sample size may influence the results found here.

When asked about the type of speech complaint, orofacial motricity (motor impulse of face and mouth) was one of the least mentioned; however, the use of bottle and/or pacifiers and/or unhealthy oral habits had relevant values in (67.5%) in relation to the total sample.

Table 5 does not bring statistically relevant p-value, however it is possible to observe an association between the altered PDL and the time parents spend with their children, less than 8 hours/day (27.4%).

**Table 5:** Time spend with child

**PDL**	**Time parents spend with their children (hours)**						**Total**
	**½ to 1**	**2 to 4**	**6 to 8**	**9 to 12**	**24**	**NR**	
Altered	21 (10.8%)	31 (16.0%)	29 (14.9%)	16 (8.2%)	16 (8.2%)	8 (4.1%)	121 (62.4%)
Non-altered	11 (5.7%)	21 (10.8%)	26 (13.4%)	7 (3.6%)	4 (2.1%)	4 (2.1%)	73 (37.6%)
Total	32 (16.5%)	52 (26.8%)	55 (28.4%)	23 (11.9%)	20 (10.3%)	12 (6.2%)	194 (100.0%)

Pearson's Chi-squared test data: Table X-squared = 5.742. df = 5. p-value = 0.3321

Caption: PDL = Language Development Protocol; NR = Not reported

Another analysis was conducted to further understand the delay in seeking speech therapy services and the time from the first complaint. We analyzed the time interval, in months, between the child's age and the first complaint and related variables such as maternal education level, paternal education level, the time parents spend with their children and the socioeconomic status only in the altered PDL.

A statistically relevant p-value was also found between this time interval and family socioeconomic status.

## DISCUSSION

The results we have found demonstrate important risk factors: firstly that most of the participants are male and that the greatest demand for the speech-therapy service comes from declared white race. They also show that the parental education level has a huge play in the search for speech therapy services, as well as that the mother's temperament affects the children’s development. Finally they show that mental health related-risks life can affect language development.

Results show that male participants prevailed in the study. And studies show that boys have slower maturation of the nervous system in boys. Therefore, we can argue that this stands for a risk factor^(10)^.

It was found that the greatest demand for the service is from the declared Caucasian race. In an American systematic review, the data collected presented a correlation between health literacy and issues such as age, race/ethnicity, years of education and cognitive function. Regarding race/ethnicity, of the 23 studies analyzed by this review, 15 had a predominantly white sample, 6 had a predominantly African-American sample, one had a predominantly Hispanic sample, and the only study conducted outside the United States reportedly had a 100% Asian sample^(16)^. These data corroborate our findings in this research, which would lead to us to believe that declared white race has greater access to health literacy when compared to others and to consider our role in adopting strategies that can minimize this discrepancy.

Children who live in an unfavorable socioeconomic situation have developmental disadvantages and are more vulnerable to language-related complications^(5)^. Parental education level plays an important role in the child's cognitive development, given that the literature establishes a correlation between a higher level of parental education level and the promotion of a positive environment for language development^(17)^.

Based on the results found in the application of both protocols, it is possible to infer that the parental education level is a risk factor. Moreover, the maternal education level has a significant influence on the development of the reading habit within the family environment, which positively interferes with the acquisition and the development of a child's language.

We sought to correlate the time interval between the age of the first complaint and the child's current age, in units of months. The critical analysis of this time interval has enabled us to observe that the older the child, the longer the time interval between the first complaint and the search for a speech therapy service. It is likely that the complaint takes place once the child enters kindergarten and starts to be compared to their peers.

Even though the p-value has no relevant statistical value, we are aware the importance of family health history and the complaint. As for the influence of family history, a study by Zambrana et al.^(18)^ shows that an integrative model of risk factors could predict the occurrence of persistent or late-onset language delay trajectories from three to five years. Therefore, the family history alone could be the deciding risk factor on the appearance of speech-related disorders in children.

However, it is noteworthy that the history of the current complaint and/or the past pathology of the individual can describe the person as a whole. Knowing the family history helps and coherently leads us to think about the next steps of care^(19)^.

When discussing the children's altered temperament, based on the parents/guardians' answers, some studies show that mental health related-risks in the first years of life can affect language development and child development as a whole; that is, the unbalance of the mental and emotional aspects of the child’s life can contribute to the emergence of difficulties in the acquisition of future skills. Since the first months of life, the child's brain plasticity is very intense. Environmental factors can aggravate psychosocial risk factors and consequently affect the healthy development of a child^(20)^.

Current research shows that temperament undergoes modulation at younger ages. Hence, the characteristics of the child's temperament may have an influence on development, but it is not possible to state that it will determine temperament change^(21)^.

One study has shown that in the interaction analysis of 10 dyads (mother-infant), the children who showed anger have mothers with lower interactive skills and needed a high percentage of exchange initiatives with no response from the child. On the other hand, the dyads that presented a higher percentage of shared activities corresponded to mothers with more interactive skills^(22)^. These studies corroborate with our findings in the sense that the mother's temperament is a factor to be considered in the development of the child.

The pre, peri and postnatal complications may impact on the acquisition of motor and cognitive skills^(23)^. Furthermore, when exposed to a psychologically unfavorable environment, they experience high-risk conditions for physical and mental health, since family characteristics are strongly associated with mental health in child development^(24)^.

The importance of the time parents spend with their children, according to current research lies in the quality of this time with the child and how this impacts on language development. That quality time spent together in fact poses as a source for children well-being. Furthermore, the less time parents spend with their children, the longer the delay in seeking speech therapy services. A statistically relevant p-value was also found between this time interval and family socioeconomic status.

This research also suggests that future clinical research and clinical work performed by health professionals, especially in PHC should consider risk factors that influence children's language development for speech-language pathology and audiology screening. Furthermore, continued studies are needed for improvements in the Brazilian children's public policy of primary health care.

Clinical applications of this study would entail how helpful to health professionals in Primary Health Care the application of these protocols would be as early speech-language screening. The early identification of language disorders might create the necessary bond for continuity of care to that specific family in a risk group.

In order to broaden the scope of the topic observed, we hope that the results will imply in the care of the participant, the creation and reorganization of public health policies among other tools that will be used to meet the population's demands and the continuity of this study is of great value to scientific research.

## CONCLUSION

The research has achieved its goals, firstly, by correlating the PIFRAL and PDL protocols and secondly by showing some of the risk factors for child development and their implications on language acquisition.

Speech-language screening is fundamental as it is the first step for many children with language development disorders. A watchful eye on behalf of the health professional is vital so that the focus is not on the pathology and its symptoms, rather on the individual as a whole and in their insertion and social, historical and cultural dimension. Early speech-language screening is also an important ally when helping create and keep a bond with these users, providing continuity of care for families in risk groups, especially.
